# Editorial: Innovative technologies and advancements in designing custom-made ornamental plants

**DOI:** 10.3389/fpls.2023.1348949

**Published:** 2023-12-12

**Authors:** Suprasanna Penna, Shri Mohan Jain

**Affiliations:** ^1^ Amity Centre for Nuclear Biotechnology, Amity Institute of Biotechnology, Amity University of Maharashtra, Mumbai, India; ^2^ Department of Agricultural Sciences, University of Helsinki, Helsinki, Finland

**Keywords:** ornamental plants, floral traits, stress tolerance, genetic modification, floriculture, omics, genetic engineering, postharvest

Ornamental plants are used in all occasions worldwide for their novel flower traits, e.g., fragrance, flower colour and shape, early flowering, plant type, stress tolerance and long shelf-life (Wani et al., 2018). The floricultural industry, worth over 50 billion Euros, thus serves to contribute to the socio-economic impact in several countries across the globe (Darras, 2020). Edible flowers due to their unique organoleptic and nutraceutical profile, have been the research focus, especially considering the phytochemicals that play a functional role in plant defense, and human health (Benvenuti and Mazzoncini, 2021). The advent of *in vitro* technologies and high volume micropropagation has been of immense value to the industry by providing high value planting material and scope for introducing novel traits of significance to floriculture industry. *In vitro* approaches such as haploidy, polyploidy, cryopreservation, somaclonal variation and genetic modification offer a great scope for the conservation and development of new elite clones. Further, *in vitro* mutagenesis and selection, doubled haploids, molecular markers, and molecular breeding enable the breeders to exploit and characterize genetic variability. The ornamental plants have been the focus of the bioeconomy, and, thus the research advancements in floricultural biotechnology and genomics approaches have added the necessary momentum for plant improvement.

Micropropagation has been well exploited commercially for large-scale plant production of ornamental plants, and in the recent years the advent of vertical and digital farming, and artificial intelligence has transformed the floriculture industry (Abdalla et al., 2022; Wani et al., 2023). Bioreactors and temporary immersion systems are now applied as a cost effective systems for large-scale production of *in vitro* propagules. Continued research into high-volume propagation systems through bioreactors and immersion systems is essential to cater to the needs of floriculture industry. Advances in genomics, nanotechnology, and gene editing are the new technologies for designing custom-made novel floral traits benefiting both the ornamental and cosmetic industries (Ahn et al., 2020; Jin et al., 2023).

Strategies of genetic modification of flowering plants for producing a variety of flower colours and other attributes related to post-harvest are now available to develop elite varieties (Ghag et al., 2022; Suprasanna and Jain, 2022). Such genetically modified varieties with novel floral attributes will offer more benefits to growers and consumers. Despite such commercial potential, only small quanta of GM ornamental plants have undergone field testing for their release into the market. Ever since the development of color-modified carnation and rose couple of decades ago, very few other ornamental varieties have been transformed and field tested and released (Boutigny et al., 2020). With the new genomics tools and genome editing methods, it is now becoming possible to precisely alter floricultural traits such as colour, early maturity, morphology, fragrance and shelf-life, by using altered biosynthetic pathways through gene incorporation, overexpression, or silencing. This Research Topic has covered the current applications of cellular and molecular approaches for understanding and improving ornamental plant species for a variety of attributes that are crucial for the floriculture industry.

Floral traits are of evolutionary and industrial significance especially higher reproductive behavior and speciation in the horticultural plants. In case of ornamental plants, floral attributes play an important role for breeding better varieties, however knowledge of their genetic control is less understood. There has been considerable interest to molecular mechanisms and markers specific to floral traits (Zhang et al., 2018; Wang et al., 2022). Li et al. conducted a mapping study to identify markers for different floral traits and their control by specific QTLs in mei (*Prunus mume*). Authors observed that QTLs were located in a narrow region of chromosome 1 and, further transcriptomic study of different genotypes indicated there were several differentially expressed genes conferring several biological functions associated with flower formation, petal color and petal number. The identified QTLs also displayed pleiotropic effects on other non-floral traits like shoot length. Characterization of such pleiotropic QTLs can be useful to gain information on the genetic architecture and regulation of floral diversity. Floral QTL mediated pleiotropic effects have shed light on budburst in *Juglans regia* L. (Bükücü et al., 2020) and shoot growth in Arabidopsis (Hiraoka et al., 2013), fitness in perennial grass (Weng et al., 2022).

Among the plant metabolites, floral volatile compounds are of great interest owing to their multitude functions (Bouwmeester et al., 2019). Unravelling the mechanism of the accumulation of floral colours and scent is essential to their functions in plants and to breed new color and scent varieties. *Syringa oblata* Lindl., a woody ornamental plant with multiple flower colors and strong fragrance, has very great commercial value. Molecular studies on the regulation of these traits are lacking, and the study by Chen et al. offers chromosome level genome assembly of *Syringa oblata* using Illumina Hiseq X-ten. The authors generated the genome size of 1.11 Gb with a contig N50 of 4.75 Mb, secured on 23 chromosomes, offering a good reference system for *Syringa oblate* transcriptome. *De novo* predictions, homology-based predictions, metabolomics and RNA sequencing data were employed to build gene models for the *S. oblata* genome. The data from the transcriptomic and metabolic studies led to novel findings about the candidate genes F3H, F3’H, 4CL and PAL, associated with the diversity in floral color. For the floral volatiles, authors found that TPS-b subfamily in association with the CYP76 family genes were the determinants. This study has offered valuable insights into the mechanistic cues underlying floral attributes of commercial significance which can be exploited in breeding better varieties.

Orchids are one of the ornamentals with a rich glory of being the fascinating plants in the plant kingdom. The most challenging aspect for their conservation and re-introduction has been the ecological dependencies including mycorrhizal fungi for their germination. Yang et al. demonstrated a technique of complexing dust like seed of orchid with mycorrhizal fungus, in to a granular form to augment higher seed germination and seedling production. Five different orchid species (*Dendrobium officinale, Tulasnella* sp.*, Dendrobium devonianum, Paphiopedilum spicerianum, Arundina graminifolia*) with different germination rates were tested and the results showed that 1% polymer water-absorbent resin added to the seed-fungus complexes of *D. officinale* seeds enhanced germination and seed viability. The study highlighted the utilization of low-cost seed-fungus complexes for orchid conservation, seed germination, and seedling productivity.

Breeding improved varieties of ornamental plants has always been the focus of horticulturists to consider novel traits, yield and stress tolerance. Biotechnological and genomics tools have empowered breeders to embark upon genetic modification of the target traits (Ghag et al., 2022). In recent years, a great deal of information has been generated on the agronomic and horticultural traits which has set the pace for targeted modification of functional traits. Jin et al. have presented a detailed overview and an update on transgenic breeding studies and, proposed a framework to be adopted in ornamental breeding. The authors showed that recombination manipulation, haploid inducer creation, clonal seed production, and reverse breeding have a great potential for use in improvement of ornamental plant species. For instance, researchers can apply the multiple anti-crossover (anti-CO) genes that limit meiotic recombination in ornamental breeding. The haploid-inducer system (similar to maize) can be used to induce maternal haploids and the method is presently applied in several plants. Use of CENH3 (CENTROMERIC HISTONE H3) induces chromosome lagging and formation of micronuclei, and hence CENH3 modification has become a potential haploid inducer system. The authors also suggested reverse breeding to generate perfectly complementing homozygous parental lines from the heterozygous plants, and that clonal seed can be developed to maintain heterozygosity of the F1 hybrids (Wang et al., 2019).

Plants with their attractive and beautiful coloured foliage and flowers are the nature’s splendour. Anthocyanins being the most common pigments offer a diverse spectrum of visible colors from red-magenta to blue-purple, and the pigment biosynthetic pathways can be genetically re-designed to yield novel floral colours for example, blue colored poinsettia (*Euphorbia pulcherrima* Willd. ex Klotzsch). Lozoya-Gloria et al. presented possibilities for genetic engineering of the anthocyanin biosynthetic pathway in *E. pulcherrima.* The authors also described in detail the pathways, *in vitro* and transformation methods required for genetic engineering of the plant. It is interesting to see that the delphinidin biosynthetic genes from other plant sources can be transformed under the transcriptional regulation of a pathway-specific promoter into *E. pulcherrima*. The authors also argue that structural or transcription factor genes may be over-expressed to yield an abundance of anthocyanins, and stable gene silencing or the genome editing of some key genes could be used to alter the color of the bract. Such experimental strategies can be significant to engineer novel attributes of commercial interest in ornamental plants.

In conclusion, this Research Topic has shed light on the approaches for improving ornamental plant species for floral and foliage related traits, which are crucial for the floriculture industry. The articles have highlighted the exploitation of biotechnological, and genomics tools for ornamental plant species. It is also suggested that such experimental platforms should help researchers to fine-tune their efforts for improving commercially important ornamental plants.

## Author contributions

SP: Conceptualization, Writing – original draft, Writing – review & editing. SJ: Conceptualization, Writing – review & editing.
